# (Dimethyl sulfoxide-κ*O*)bis­(thio­semicarbazide-κ^2^ *N* ^1^,*S*)zinc dipicrate dimethyl sulfoxide solvate monohydrate

**DOI:** 10.1107/S160053681005378X

**Published:** 2011-01-08

**Authors:** R. Shanthakumari, Ramu Hema, K. Ramamurthy, Balasubramnian Sridar, Helen Stoeckli-Evans

**Affiliations:** a Department of Physics, Government Arts College for Women, Pudukottai 622 001, Tamil Nadu, India; b Department of Physics, Seethalakshmi Ramaswami College (Autonomous), Tiruchirappalli 620 002, India; c Crystal Growth and Thin Film Laboratory, School of Physics, Bharathidasan University, Tiruchirapalli 620 024, India; d Laboratory of Crystallography, Indian Institute of Chemical Technology, Hyderabad 500 007, India; e Institute of Physics, University of Neuchâtel, Rue Emile-Argand 11, CH-2000, Neuchâtel, Switzerland

## Abstract

The title complex, [Zn(CH_5_N_3_S)_2_(C_2_H_6_OS)](C_6_H_2_N_3_O_7_)_2_·C_2_H_6_OS·H_2_O, is composed of a [Zn(thio­semi­carbazide)_2_(DMSO)]^2+^ cation (where DMSO is dimethyl sulfoxide), and two picrate anions. In the asymmetric unit, there is also a solvent mol­ecule of DMSO and a water mol­ecule of crystallization. In the cation, the Zn^II^ atom is five-coordinated in a distorted square–pyramidal geometry. It coordinates to the O atom of a DMSO mol­ecule and to the S and one N atom of two thio­semicarbazide mol­ecules, which behave as bidentate ligands coordinating in a *trans* arrangement. In the crystal, a number of N—H⋯O, O—H⋯O and N—H⋯S hydrogen bonds link the mol­ecules into two-dimensional networks. These networks are further linked *via* weak C—H⋯O inter­actions, forming a three-dimensional arrangement. Positional disorder in one methyl group of the coordinated DMSO molecule and in the two picrate anions was observed.

## Related literature

For the biological activity of thio­semicarbazides, see: Gowda & Mahadevappa (1977) ▶; Pillai *et al.* (1977 ▶). For the use of thio­semicarbazide as a masking agent, see: Kirkbright & Taddia (1978 ▶). For the crystal structure of a similar five-coordinate zinc(II)–thio­semicarbazide complex, see: Babb *et al.* (2003 ▶). For a description of five-coordinate metal atoms, see: Addison *et al.* (1984 ▶).
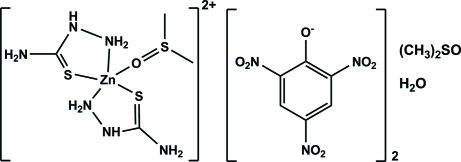


## Experimental

### 

#### Crystal data

[Zn(CH_5_N_3_S)_2_(C_2_H_6_OS)](C_6_H_2_N_3_O_7_)_2_·C_2_H_6_OS·H_2_O
*M* *_r_* = 878.13
Triclinic, 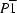

*a* = 10.8762 (11) Å
*b* = 11.2559 (12) Å
*c* = 14.4859 (15) Å
α = 81.124 (2)°
β = 77.063 (2)°
γ = 81.168 (2)°
*V* = 1694.6 (3) Å^3^
*Z* = 2
Mo *K*α radiation
μ = 1.06 mm^−1^
*T* = 294 K
0.24 × 0.24 × 0.20 mm

#### Data collection

Bruker SMART APEX CCD area-detector diffractometer
18781 measured reflections
7719 independent reflections
6573 reflections with *I* > 2σ(*I*)
*R*_int_ = 0.021

#### Refinement

*R*[*F* ^2^ > 2σ(*F* ^2^)] = 0.034
*wR*(*F* ^2^) = 0.095
*S* = 1.05
7719 reflections
546 parameters
6 restraints
H atoms treated by a mixture of independent and constrained refinement
Δρ_max_ = 0.54 e Å^−3^
Δρ_min_ = −0.37 e Å^−3^

### 

Data collection: *SMART* (Bruker, 2007 ▶); cell refinement: *SAINT* (Bruker, 2007 ▶); data reduction: *SAINT*; program(s) used to solve structure: *SHELXS97* (Sheldrick, 2008 ▶); program(s) used to refine structure: *SHELXL97* (Sheldrick, 2008 ▶); molecular graphics: *PLATON* (Spek, 2009 ▶); software used to prepare material for publication: *SHELXL97* and *PLATON*.

## Supplementary Material

Crystal structure: contains datablocks I, global. DOI: 10.1107/S160053681005378X/hg2775sup1.cif

Structure factors: contains datablocks I. DOI: 10.1107/S160053681005378X/hg2775Isup2.hkl

Additional supplementary materials:  crystallographic information; 3D view; checkCIF report

## Figures and Tables

**Table 1:** Hydrogen-bond geometry (Å, °)

*D*—H⋯*A*	*D*—H	H⋯*A*	*D*⋯*A*	*D*—H⋯*A*
N1—H1*NA*⋯O1*W*^i^	0.87 (2)	2.20 (2)	2.974 (3)	148 (2)
N2—H*N*2⋯O15*A*^i^	0.80 (2)	2.41 (3)	3.01 (2)	133 (2)
N2—H*N*2⋯O16^i^	0.80 (2)	1.96 (2)	2.693 (2)	152 (2)
N3—H3*NA*⋯O10^i^	0.87 (3)	2.44 (3)	3.166 (2)	141 (2)
N3—H3*NA*⋯O16^i^	0.87 (3)	2.03 (3)	2.790 (3)	146 (3)
N3—H3*NB*⋯O5^ii^	0.77 (2)	2.24 (2)	3.004 (3)	173 (2)
N5—H5*N*⋯O7^iii^	0.84 (2)	2.37 (2)	3.007 (3)	133 (2)
N5—H5*N*⋯O9*A*^iii^	0.84 (2)	1.94 (3)	2.698 (17)	150 (2)
N4—H4*NA*⋯O2^iii^	0.88 (2)	2.20 (2)	2.933 (3)	141 (2)
N4—H4*NB*⋯S1^iv^	0.84 (3)	2.63 (3)	3.457 (2)	170 (2)
N6—H6*NA*⋯O6^iii^	0.82 (3)	2.41 (3)	3.081 (3)	140 (2)
N6—H6*NA*⋯O9*A*^iii^	0.82 (3)	2.05 (5)	2.76 (3)	145 (3)
N6—H6*NB*⋯O11^v^	0.82 (3)	2.22 (3)	3.034 (3)	172 (3)
O1*W*—H1*WA*⋯O8	0.819 (19)	2.27 (2)	3.059 (3)	163 (3)
O1*W*—H1*WB*⋯O2	0.80 (2)	2.02 (2)	2.806 (3)	171 (3)
C6—H6*B*⋯O2^iii^	0.96	2.52	3.347 (3)	145
C8—H8⋯O4^vi^	0.93	2.47	3.390 (3)	168
C4*A*—H4*A*2⋯O1*W*	0.96	2.48	3.430 (7)	170

Symmetry codes: (i) 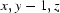; (ii) 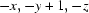; (iii) 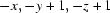; (iv) 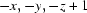; (v) 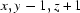; (vi) 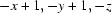.

**Fig. 1.**
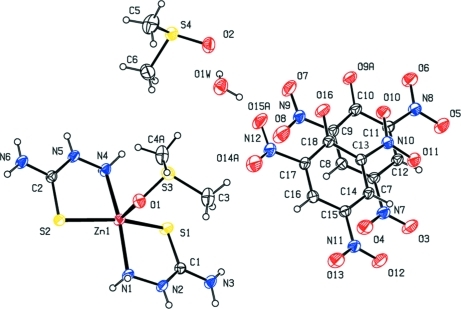
View of the asymmetric unit of the title compound showing the atom-labelling scheme. Displacement ellipsoids are drawn at the 50% probability level. Only the principal components of the disordered atoms are shown.

**Fig. 2.**
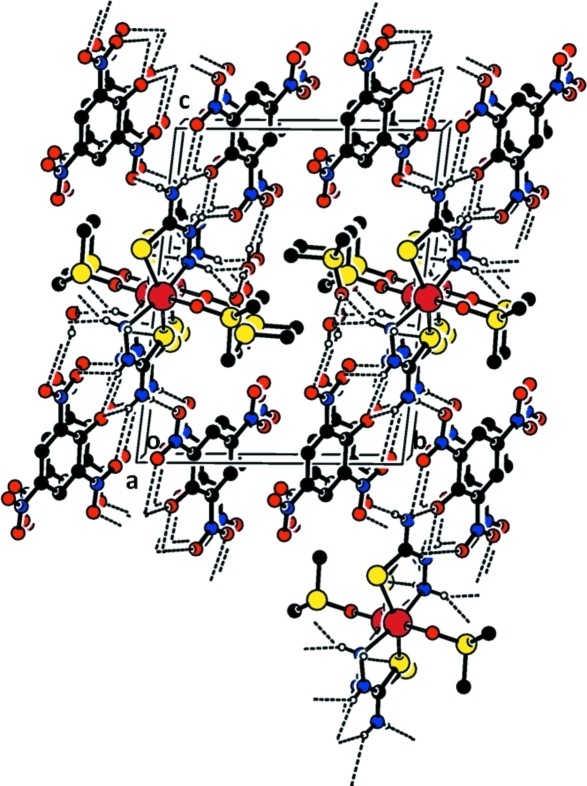
A view along the a axis of the crystal packing of the title compound. The hydrogen bonds are shown as dashed lines [details are given in Table 1; C-bound H-atoms have been omitted for clarity].

## supplementary crystallographic information

### Comment

Thosemicarbazide, a well known chelating agent, is used to characterize aldehydes, ketones, and polysaccharides. Some thiosemicarbazide derivatives are potential anti-tumor, anti-hypertensive agents, and are active against influenza, protozoa and smallpox (Gowda & Mahadevappa, 1977; Pillai *et al.*, 1977). Thiosemicarbazide is also used as a masking agent to minimize interference from metals such as copper, nickel and platinum in the determination of arsenic by atomic absorption methods (Kirkbright & Taddia, 1978). The conformational preferences of thiosemicarbazide in metal-complex formation are therefore of some interest. The reaction of zinc chloride with thiosemicarbazide in the presence of picric acid gave a yellow powder that was recrystallized using DMSO. This lead to the formation of yellow crystals of the title compoud, a DMSO-water solvate.

The molecular structure of the title complex is illustrated in Fig. 1. In the cation [Zn(thiosemicarbazide)_2_(DMSO)]^2+^ the thiosemicarbazide ligands coordinate in a bidentate mode, bonding to atom Zn1 through atoms S1, N1 and S2, N4, in a *trans* arrangment. Atom Zn1 is also coordinated to a DMSO molecule through the O-atom, O1. The zinc atom has a distorted square pyramidal coordination sphere with a τ value of 0.17 [τ = 0 for square pyramidal, τ = 1 for trigonal bipyramidal; Addison *et al.*, 1984]. The bond distances are comparable to those in a related penta-coordinated complex, (Citraconato-*O*)-bis(thiosemicarbazide-N,*S*)-zinc(II) monohydrate (Babb *et al.*, 2003). Interestingly, here the thiosemicarbazide ligands are in a *cis* disposition, and the zinc coordination sphere has a τ value of 0.72, hence it can be described as a distorted trigonal bipyramid.

In the crystal a sheet-like network is formed, propagating in the *ac*-plane, as a result of a number of intermolecular N—H···O, O—H···O and N—H···S hydrogen bonds. These sheets are then linked *via* weak C—H···O interactions to form a three-dimensional arrangement (Table 1 and Fig. 2).

### Experimental

A mixture of supersaturated solutions of thiosemicarbazide, picric acid and zinc chloride were added in the molar ratio of 1:1:1 (0.9 g: 2.5 g: 2.8 g). The calculated amount of thiosemicarbazide and zinc chloride were dissolved in distilled water and picric acid dissolved in acetone was added. Within a few minutes, the solution became turbid. The reaction was ensured with continuous stirring and after 1 h a yellow product was deposited at the bottom of the beaker, it was filtered off and dried. This yellow solid was recrystallized from DMSO to afford yellow block-like crystals of the title compound (yield: 4 g, 66.6%)

### Refinement

There is a certain positional disorder in one of the methyl groups of the coordinated DMSO molecule, and in the two picrate anions. Methyl C4 was refined with occupanices of C4A/C4B = 0.5/0.5, with C—S distance restraints of 1.76 (2) Å and their ADP's were made equal to those of atom C3. O-atom O9 in one of the picrate anions was refined with occupancies of O9A/O9B = 0.56 (8)/0.44 (8), while O-atoms O14 and O15 of a NO_2_ group in the second picrate anion where refined with occupancies of O14A/O14B = O15A/O15B = 0.67 (3)/0.33 (7). There is a short O13···O13^i^ contact involving a NO_2_ O-atom [symmetry code (i) = -*x* + 2, -*y* + 1, -*z*]. This contact was refined with a distance restraint of 2.95 (3) Å. The NH_2_ and NH H atoms were located in difference electron density maps and were freely refined. The water molecule H-atoms could also be located in a difference electron density map and were refined with distance restraints of 0.84 (2) Å. The C-bound H atoms were included in calculated positions and treated as riding atoms; C—H = 0.93 and 0.96 Å for CH and methyl H-atoms, respectively, with *U*_iso_(H) = k ×*U*_eq_(C), where k =1.5 for methyl H-atoms and k = 1.2 for all other H-atoms.

### Crystal data

**Table:**

[Zn(CH_5_N_3_S)_2_(C_2_H_6_OS)](C_6_H_2_N_3_O_7_)_2_·C_2_H_6_OS·H_2_O	*Z* = 2
*M**_r_* = 878.13	*F*(000) = 900
Triclinic, *P*1	*D*_x_ = 1.721 Mg m^−^^3^
Hall symbol: -P 1	Melting point: 469 K
*a* = 10.8762 (11) Å	Mo *K*α radiation, λ = 0.71073 Å
*b* = 11.2559 (12) Å	Cell parameters from 5926 reflections
*c* = 14.4859 (15) Å	θ = 1.9–25.0°
α = 81.124 (2)°	µ = 1.06 mm^−^^1^
β = 77.063 (2)°	*T* = 294 K
γ = 81.168 (2)°	Block, yellow
*V* = 1694.6 (3) Å^3^	0.24 × 0.24 × 0.20 mm

### Data collection

**Table:**

Bruker SMART APEX CCD area-detector diffractometer	6573 reflections with *I* > 2σ(*I*)
Radiation source: fine-focus sealed tube	*R*_int_ = 0.021
graphite	θ_max_ = 28.0°, θ_min_ = 1.9°
ω scans	*h* = −14→13
18781 measured reflections	*k* = −14→14
7719 independent reflections	*l* = −18→18

### Refinement

**Table:**

Refinement on *F*^2^	Primary atom site location: structure-invariant direct methods
Least-squares matrix: full	Secondary atom site location: difference Fourier map
*R*[*F*^2^ > 2σ(*F*^2^)] = 0.034	Hydrogen site location: inferred from neighbouring sites
*wR*(*F*^2^) = 0.095	H atoms treated by a mixture of independent and constrained refinement
*S* = 1.05	*w* = 1/[σ^2^(*F*_o_^2^) + (0.0559*P*)^2^ + 0.4578*P*] where *P* = (*F*_o_^2^ + 2*F*_c_^2^)/3
7719 reflections	(Δ/σ)_max_ = 0.001
546 parameters	Δρ_max_ = 0.54 e Å^−^^3^
6 restraints	Δρ_min_ = −0.37 e Å^−^^3^

### Special details

**Table:**

Geometry. Bond distances, angles *etc*. have been calculated using the rounded fractional coordinates. All su's are estimated from the variances of the (full) variance-covariance matrix. The cell e.s.d.'s are taken into account in the estimation of distances, angles and torsion angles
Refinement. Refinement of *F*^2^ against ALL reflections. The weighted *R*-factor *wR* and goodness of fit *S* are based on *F*^2^, conventional *R*-factors *R* are based on *F*, with *F* set to zero for negative *F*^2^. The threshold expression of *F*^2^ > σ(*F*^2^) is used only for calculating *R*-factors(gt) *etc*. and is not relevant to the choice of reflections for refinement. *R*-factors based on *F*^2^ are statistically about twice as large as those based on *F*, and *R*- factors based on ALL data will be even larger.

### Fractional atomic coordinates and isotropic or equivalent isotropic displacement parameters (Å^2^)

**Table:**

	*x*	*y*	*z*	*U*_iso_*/*U*_eq_	Occ. (<1)
Zn1	0.26667 (2)	0.03164 (2)	0.50488 (1)	0.0319 (1)	
S1	0.14900 (4)	0.05204 (5)	0.38255 (3)	0.0384 (1)	
S2	0.32467 (5)	−0.06515 (5)	0.64778 (3)	0.0377 (1)	
S3	0.33061 (5)	0.30032 (5)	0.44415 (4)	0.0427 (2)	
O1	0.37531 (13)	0.16931 (13)	0.47872 (10)	0.0403 (4)	
N1	0.39527 (17)	−0.08894 (18)	0.41352 (11)	0.0353 (5)	
N2	0.37816 (15)	−0.06488 (16)	0.31838 (11)	0.0356 (5)	
N3	0.25503 (19)	−0.00172 (19)	0.20917 (12)	0.0425 (6)	
N4	0.10145 (16)	0.11037 (18)	0.60253 (11)	0.0357 (5)	
N5	0.12502 (16)	0.10238 (17)	0.69507 (11)	0.0392 (5)	
N6	0.23281 (19)	0.0216 (2)	0.81157 (13)	0.0464 (6)	
C1	0.26914 (17)	−0.01021 (16)	0.29808 (13)	0.0304 (5)	
C2	0.22014 (17)	0.02636 (17)	0.72219 (13)	0.0323 (5)	
C3	0.3683 (3)	0.3103 (3)	0.31805 (18)	0.0625 (8)	
C4A	0.4290 (6)	0.3825 (6)	0.4800 (4)	0.0625 (8)	0.500
C4B	0.4667 (6)	0.3781 (6)	0.4444 (4)	0.0625 (8)	0.500
O10	0.37612 (15)	0.93032 (16)	0.00119 (11)	0.0523 (5)	
O11	0.46364 (16)	0.86421 (16)	−0.13237 (11)	0.0530 (5)	
O12	0.83363 (17)	0.59567 (19)	−0.17971 (11)	0.0648 (6)	
O13	0.95110 (17)	0.5539 (2)	−0.07638 (13)	0.0719 (7)	
O14A	0.7817 (12)	0.655 (3)	0.2427 (8)	0.083 (4)	0.67 (7)
O15A	0.604 (2)	0.7437 (19)	0.2841 (6)	0.074 (3)	0.67 (7)
O16	0.48564 (19)	0.8581 (2)	0.14820 (12)	0.0727 (7)	
N10	0.46168 (16)	0.86917 (15)	−0.04741 (12)	0.0373 (5)	
N11	0.85340 (17)	0.60065 (17)	−0.10083 (12)	0.0432 (5)	
N12	0.6796 (2)	0.69437 (19)	0.22324 (13)	0.0535 (7)	
C13	0.56306 (18)	0.79722 (17)	−0.00584 (13)	0.0335 (5)	
C14	0.65550 (18)	0.73364 (17)	−0.06708 (13)	0.0344 (5)	
C15	0.75575 (18)	0.66393 (18)	−0.03405 (13)	0.0355 (5)	
C16	0.76483 (19)	0.65359 (18)	0.06125 (14)	0.0384 (6)	
C17	0.6701 (2)	0.71427 (19)	0.12300 (13)	0.0386 (6)	
C18	0.5638 (2)	0.79503 (19)	0.09423 (14)	0.0393 (6)	
O15B	0.575 (3)	0.709 (4)	0.2800 (14)	0.070 (5)	0.33 (7)
O14B	0.759 (4)	0.607 (4)	0.2468 (12)	0.076 (5)	0.33 (7)
O3	0.35471 (17)	0.59850 (19)	−0.20375 (12)	0.0616 (6)	
O4	0.47002 (16)	0.55050 (18)	−0.09782 (13)	0.0644 (6)	
O5	−0.02160 (17)	0.8604 (2)	−0.15007 (12)	0.0684 (7)	
O6	−0.10969 (17)	0.91940 (19)	−0.01512 (12)	0.0648 (6)	
O7	0.12595 (19)	0.7413 (2)	0.26100 (12)	0.0690 (7)	
O8	0.3023 (2)	0.6364 (2)	0.22031 (14)	0.0917 (9)	
O9A	0.002 (3)	0.849 (3)	0.1297 (7)	0.059 (4)	0.56 (8)
N7	0.37299 (16)	0.59932 (17)	−0.12325 (12)	0.0422 (5)	
N8	−0.02350 (16)	0.86148 (17)	−0.06498 (12)	0.0432 (6)	
N9	0.20614 (17)	0.69458 (17)	0.20037 (12)	0.0417 (5)	
C7	0.27455 (18)	0.66079 (18)	−0.05566 (13)	0.0349 (5)	
C8	0.28479 (17)	0.64976 (17)	0.03925 (14)	0.0352 (5)	
C9	0.19018 (18)	0.70787 (18)	0.10207 (13)	0.0356 (6)	
C10	0.0773 (2)	0.7806 (2)	0.07608 (14)	0.0434 (6)	
C11	0.07928 (18)	0.78936 (18)	−0.02508 (14)	0.0367 (6)	
C12	0.17383 (18)	0.73076 (18)	−0.08814 (13)	0.0355 (5)	
O9B	−0.0239 (12)	0.803 (4)	0.1378 (10)	0.047 (3)	0.44 (8)
S4	−0.00366 (5)	0.64929 (5)	0.61310 (4)	0.0427 (2)	
O2	0.03790 (16)	0.69781 (16)	0.50943 (11)	0.0524 (5)	
C5	0.1098 (3)	0.6799 (3)	0.6734 (2)	0.0726 (11)	
C6	0.0343 (4)	0.4902 (2)	0.6228 (2)	0.0740 (12)	
O1W	0.29788 (19)	0.67515 (18)	0.42553 (14)	0.0576 (6)	
H1NA	0.385 (2)	−0.164 (2)	0.4348 (18)	0.049 (7)*	
H1NB	0.465 (3)	−0.076 (2)	0.4097 (15)	0.047 (7)*	
HN2	0.429 (2)	−0.097 (2)	0.2780 (15)	0.039 (6)*	
H3A	0.45390	0.27340	0.29780	0.0940*	
H3B	0.36070	0.39390	0.29120	0.0940*	
H3C	0.31090	0.26890	0.29680	0.0940*	
H3NA	0.312 (3)	−0.043 (2)	0.1701 (19)	0.053 (7)*	
H3NB	0.192 (2)	0.030 (2)	0.1965 (17)	0.041 (6)*	
H5N	0.074 (2)	0.139 (2)	0.7368 (16)	0.037 (6)*	
H4NA	0.080 (2)	0.187 (2)	0.5829 (18)	0.050 (7)*	
H4NB	0.035 (3)	0.077 (2)	0.6116 (18)	0.050 (7)*	
H6NA	0.179 (3)	0.061 (2)	0.8477 (19)	0.054 (7)*	
H6NB	0.290 (3)	−0.026 (2)	0.8302 (18)	0.050 (7)*	
H4B1	0.47740	0.37480	0.50880	0.0940*	0.500
H4B2	0.45180	0.46110	0.41740	0.0940*	0.500
H4B3	0.54210	0.33900	0.40720	0.0940*	0.500
H4A1	0.42610	0.36100	0.54720	0.0940*	0.500
H4A2	0.40120	0.46750	0.46740	0.0940*	0.500
H4A3	0.51460	0.36480	0.44540	0.0940*	0.500
H16	0.83350	0.60660	0.08270	0.0460*	
H14	0.65030	0.73770	−0.13070	0.0410*	
H8	0.35460	0.60380	0.05970	0.0420*	
H12	0.16980	0.73830	−0.15230	0.0430*	
H5A	0.10470	0.76580	0.67340	0.1090*	
H5B	0.19340	0.64940	0.64160	0.1090*	
H5C	0.09310	0.64110	0.73800	0.1090*	
H6A	0.12030	0.47020	0.58990	0.1110*	
H6B	−0.02230	0.45630	0.59490	0.1110*	
H6C	0.02570	0.45780	0.68890	0.1110*	
H1WA	0.305 (3)	0.679 (3)	0.3677 (13)	0.079 (10)*	
H1WB	0.2231 (18)	0.688 (3)	0.445 (2)	0.079 (11)*	

### Atomic displacement parameters (Å^2^)

**Table:**

	*U*^11^	*U*^22^	*U*^33^	*U*^12^	*U*^13^	*U*^23^
Zn1	0.0304 (1)	0.0402 (1)	0.0272 (1)	−0.0032 (1)	−0.0084 (1)	−0.0087 (1)
S1	0.0272 (2)	0.0551 (3)	0.0363 (2)	0.0046 (2)	−0.0122 (2)	−0.0176 (2)
S2	0.0387 (2)	0.0412 (3)	0.0309 (2)	0.0088 (2)	−0.0099 (2)	−0.0067 (2)
S3	0.0398 (3)	0.0435 (3)	0.0471 (3)	0.0000 (2)	−0.0169 (2)	−0.0063 (2)
O1	0.0408 (7)	0.0413 (7)	0.0431 (8)	−0.0097 (6)	−0.0171 (6)	−0.0016 (6)
N1	0.0323 (9)	0.0488 (10)	0.0277 (8)	0.0042 (7)	−0.0144 (6)	−0.0107 (7)
N2	0.0304 (8)	0.0515 (10)	0.0240 (7)	0.0058 (7)	−0.0072 (6)	−0.0102 (7)
N3	0.0393 (10)	0.0588 (11)	0.0301 (8)	0.0083 (8)	−0.0152 (8)	−0.0103 (8)
N4	0.0290 (8)	0.0517 (10)	0.0263 (8)	0.0013 (7)	−0.0105 (6)	−0.0038 (7)
N5	0.0348 (9)	0.0560 (10)	0.0254 (8)	0.0115 (8)	−0.0103 (7)	−0.0117 (7)
N6	0.0416 (10)	0.0673 (13)	0.0288 (8)	0.0150 (9)	−0.0145 (8)	−0.0121 (8)
C1	0.0297 (8)	0.0333 (9)	0.0305 (8)	−0.0034 (7)	−0.0099 (7)	−0.0060 (7)
C2	0.0287 (8)	0.0398 (10)	0.0285 (9)	−0.0011 (7)	−0.0083 (7)	−0.0039 (7)
C3	0.0726 (16)	0.0699 (13)	0.0501 (13)	−0.0240 (12)	−0.0251 (11)	0.0108 (11)
C4A	0.0726 (16)	0.0699 (13)	0.0501 (13)	−0.0240 (12)	−0.0251 (11)	0.0108 (11)
C4B	0.0726 (16)	0.0699 (13)	0.0501 (13)	−0.0240 (12)	−0.0251 (11)	0.0108 (11)
O10	0.0416 (8)	0.0646 (10)	0.0461 (9)	0.0187 (7)	−0.0124 (7)	−0.0132 (7)
O11	0.0544 (9)	0.0681 (11)	0.0376 (8)	0.0168 (8)	−0.0242 (7)	−0.0121 (7)
O12	0.0578 (10)	0.0959 (14)	0.0380 (9)	0.0225 (10)	−0.0137 (7)	−0.0260 (9)
O13	0.0516 (10)	0.0996 (15)	0.0582 (11)	0.0363 (10)	−0.0204 (8)	−0.0229 (10)
O14A	0.088 (4)	0.106 (10)	0.058 (3)	0.038 (5)	−0.046 (2)	−0.023 (4)
O15A	0.078 (5)	0.108 (6)	0.035 (2)	0.028 (4)	−0.027 (3)	−0.026 (3)
O16	0.0752 (12)	0.0983 (15)	0.0355 (8)	0.0452 (11)	−0.0187 (8)	−0.0257 (9)
N10	0.0357 (8)	0.0421 (9)	0.0349 (8)	0.0023 (7)	−0.0136 (7)	−0.0050 (7)
N11	0.0393 (9)	0.0509 (10)	0.0361 (9)	0.0071 (8)	−0.0073 (7)	−0.0084 (7)
N12	0.0645 (13)	0.0628 (12)	0.0340 (9)	0.0129 (10)	−0.0239 (9)	−0.0088 (8)
C13	0.0337 (9)	0.0361 (9)	0.0312 (9)	0.0037 (7)	−0.0126 (7)	−0.0048 (7)
C14	0.0367 (10)	0.0398 (10)	0.0275 (8)	−0.0008 (8)	−0.0102 (7)	−0.0056 (7)
C15	0.0341 (9)	0.0400 (10)	0.0318 (9)	0.0021 (8)	−0.0081 (7)	−0.0076 (7)
C16	0.0383 (10)	0.0418 (10)	0.0364 (10)	0.0046 (8)	−0.0165 (8)	−0.0050 (8)
C17	0.0453 (11)	0.0437 (11)	0.0279 (9)	0.0027 (9)	−0.0142 (8)	−0.0058 (8)
C18	0.0416 (11)	0.0446 (11)	0.0305 (9)	0.0067 (8)	−0.0108 (8)	−0.0084 (8)
O15B	0.066 (7)	0.122 (12)	0.021 (4)	0.010 (8)	−0.012 (4)	−0.019 (5)
O14B	0.106 (11)	0.075 (11)	0.039 (4)	0.041 (9)	−0.036 (5)	−0.008 (5)
O3	0.0527 (10)	0.0909 (13)	0.0397 (9)	0.0133 (9)	−0.0087 (7)	−0.0270 (8)
O4	0.0427 (9)	0.0877 (13)	0.0552 (10)	0.0275 (9)	−0.0122 (8)	−0.0176 (9)
O5	0.0597 (11)	0.1050 (15)	0.0401 (9)	0.0329 (10)	−0.0282 (8)	−0.0239 (9)
O6	0.0496 (10)	0.0909 (14)	0.0490 (9)	0.0321 (9)	−0.0181 (8)	−0.0228 (9)
O7	0.0631 (11)	0.1053 (15)	0.0325 (8)	0.0230 (10)	−0.0140 (8)	−0.0171 (9)
O8	0.0740 (13)	0.147 (2)	0.0503 (11)	0.0524 (14)	−0.0364 (10)	−0.0318 (12)
O9A	0.057 (6)	0.078 (9)	0.0335 (19)	0.029 (6)	−0.012 (2)	−0.018 (3)
N7	0.0368 (9)	0.0498 (10)	0.0376 (9)	0.0026 (7)	−0.0047 (7)	−0.0104 (7)
N8	0.0364 (9)	0.0570 (11)	0.0379 (9)	0.0079 (8)	−0.0166 (7)	−0.0116 (8)
N9	0.0425 (9)	0.0521 (10)	0.0323 (8)	0.0006 (8)	−0.0153 (7)	−0.0064 (7)
C7	0.0296 (9)	0.0408 (10)	0.0336 (9)	0.0004 (7)	−0.0059 (7)	−0.0085 (7)
C8	0.0298 (9)	0.0397 (10)	0.0359 (9)	0.0020 (7)	−0.0112 (7)	−0.0040 (8)
C9	0.0351 (10)	0.0435 (10)	0.0294 (9)	−0.0008 (8)	−0.0105 (7)	−0.0065 (7)
C10	0.0377 (10)	0.0595 (13)	0.0321 (10)	0.0103 (9)	−0.0117 (8)	−0.0127 (9)
C11	0.0325 (9)	0.0463 (11)	0.0328 (9)	0.0055 (8)	−0.0138 (8)	−0.0092 (8)
C12	0.0337 (9)	0.0447 (10)	0.0295 (9)	−0.0007 (8)	−0.0097 (7)	−0.0088 (8)
O9B	0.037 (3)	0.069 (9)	0.033 (3)	0.011 (4)	−0.007 (2)	−0.020 (4)
S4	0.0414 (3)	0.0479 (3)	0.0403 (3)	−0.0013 (2)	−0.0136 (2)	−0.0069 (2)
O2	0.0545 (9)	0.0617 (10)	0.0409 (8)	−0.0040 (8)	−0.0163 (7)	0.0005 (7)
C5	0.083 (2)	0.093 (2)	0.0590 (16)	−0.0320 (17)	−0.0335 (15)	−0.0139 (14)
C6	0.122 (3)	0.0469 (14)	0.0635 (16)	−0.0030 (15)	−0.0447 (17)	−0.0084 (12)
O1W	0.0559 (11)	0.0734 (12)	0.0472 (10)	−0.0066 (9)	−0.0200 (9)	−0.0065 (9)

### Geometric parameters (Å, °)

**Table:**

Zn1—S1	2.3728 (5)	N3—H3NB	0.77 (2)
Zn1—S2	2.3554 (6)	N4—H4NA	0.88 (2)
Zn1—O1	2.0302 (15)	N4—H4NB	0.84 (3)
Zn1—N1	2.1662 (19)	N5—H5N	0.84 (2)
Zn1—N4	2.1852 (18)	N6—H6NA	0.82 (3)
S1—C1	1.7214 (19)	N6—H6NB	0.82 (3)
S2—C2	1.718 (2)	N10—C13	1.452 (3)
S3—O1	1.5240 (16)	N11—C15	1.446 (3)
S3—C3	1.770 (3)	N12—C17	1.459 (3)
S3—C4A	1.724 (7)	N7—C7	1.448 (3)
S3—C4B	1.833 (7)	N8—C11	1.452 (3)
S4—C5	1.764 (3)	N9—C9	1.455 (3)
S4—C6	1.766 (2)	C3—H3C	0.9600
S4—O2	1.5088 (17)	C3—H3A	0.9600
O10—N10	1.223 (2)	C3—H3B	0.9600
O11—N10	1.236 (2)	C4A—H4A3	0.9600
O12—N11	1.220 (2)	C4A—H4A1	0.9600
O13—N11	1.213 (3)	C4A—H4A2	0.9600
O14A—N12	1.208 (17)	C4B—H4B3	0.9600
O14B—N12	1.26 (4)	C4B—H4B2	0.9600
O15A—N12	1.203 (16)	C4B—H4B1	0.9600
O15B—N12	1.25 (3)	C13—C14	1.373 (3)
O16—C18	1.235 (3)	C13—C18	1.448 (3)
O3—N7	1.228 (2)	C14—C15	1.374 (3)
O4—N7	1.219 (3)	C15—C16	1.392 (3)
O5—N8	1.230 (2)	C16—C17	1.374 (3)
O6—N8	1.217 (3)	C17—C18	1.454 (3)
O7—N9	1.211 (3)	C14—H14	0.9300
O8—N9	1.214 (3)	C16—H16	0.9300
O9A—C10	1.25 (3)	C7—C8	1.389 (3)
O9B—C10	1.272 (19)	C7—C12	1.376 (3)
O1W—H1WB	0.80 (2)	C8—C9	1.368 (3)
O1W—H1WA	0.819 (19)	C9—C10	1.457 (3)
N1—N2	1.411 (2)	C10—C11	1.449 (3)
N2—C1	1.322 (3)	C11—C12	1.373 (3)
N3—C1	1.318 (3)	C8—H8	0.9300
N4—N5	1.407 (2)	C12—H12	0.9300
N5—C2	1.328 (3)	C5—H5B	0.9600
N6—C2	1.325 (3)	C5—H5C	0.9600
N1—H1NB	0.78 (3)	C5—H5A	0.9600
N1—H1NA	0.87 (2)	C6—H6C	0.9600
N2—HN2	0.80 (2)	C6—H6A	0.9600
N3—H3NA	0.87 (3)	C6—H6B	0.9600
			
S1—Zn1—S2	152.59 (2)	S3—C4A—H4A3	109.00
S1—Zn1—O1	108.71 (4)	C4B—C4A—H4A1	137.00
S1—Zn1—N1	82.09 (5)	S3—C4A—H4A2	110.00
S1—Zn1—N4	91.39 (5)	C4B—C4A—H4B2	54.00
S2—Zn1—O1	98.68 (4)	S3—C4A—H4B1	141.00
S2—Zn1—N1	95.55 (5)	S3—C4A—H4B2	106.00
S2—Zn1—N4	82.99 (5)	H4B2—C4A—H4A3	71.00
O1—Zn1—N1	97.42 (7)	H4A1—C4A—H4A2	109.00
O1—Zn1—N4	99.60 (7)	H4A1—C4A—H4A3	109.00
N1—Zn1—N4	162.94 (7)	H4A2—C4A—H4A3	109.00
Zn1—S1—C1	97.02 (7)	C4B—C4A—H4B1	93.00
Zn1—S2—C2	96.46 (7)	H4B1—C4A—H4A2	109.00
O1—S3—C3	104.91 (12)	H4B1—C4A—H4A3	65.00
O1—S3—C4A	103.8 (2)	H4B2—C4A—H4A1	142.00
O1—S3—C4B	102.5 (2)	C4B—C4A—H4A2	97.00
C3—S3—C4A	108.6 (2)	H4B1—C4A—H4B2	108.00
C3—S3—C4B	91.3 (2)	S3—C4B—H4B3	109.00
C5—S4—C6	97.69 (17)	S3—C4B—H4A3	136.00
O2—S4—C5	106.85 (12)	S3—C4B—H4A2	93.00
O2—S4—C6	107.24 (12)	H4B2—C4B—H4B3	110.00
Zn1—O1—S3	124.56 (9)	S3—C4B—H4B1	109.00
H1WA—O1W—H1WB	104 (3)	S3—C4B—H4B2	109.00
Zn1—N1—N2	112.09 (13)	H4B2—C4B—H4A3	112.00
N1—N2—C1	121.00 (16)	H4B3—C4B—H4A2	150.00
Zn1—N4—N5	111.38 (13)	H4A2—C4B—H4A3	129.00
N4—N5—C2	121.32 (17)	C4A—C4B—H4B2	96.00
Zn1—N1—H1NB	108.9 (16)	C4A—C4B—H4B3	152.00
H1NA—N1—H1NB	112 (2)	C4A—C4B—H4A3	120.00
N2—N1—H1NA	109.0 (16)	H4B1—C4B—H4B2	110.00
N2—N1—H1NB	103.8 (16)	H4B1—C4B—H4B3	109.00
Zn1—N1—H1NA	110.6 (16)	H4B1—C4B—H4A2	79.00
C1—N2—HN2	118.8 (16)	H4B1—C4B—H4A3	71.00
N1—N2—HN2	118.9 (16)	N10—C13—C18	120.67 (17)
C1—N3—H3NB	118.7 (18)	C14—C13—C18	123.49 (18)
H3NA—N3—H3NB	123 (3)	N10—C13—C14	115.84 (16)
C1—N3—H3NA	117.8 (19)	C13—C14—C15	119.81 (17)
Zn1—N4—H4NA	111.4 (16)	N11—C15—C16	119.95 (18)
Zn1—N4—H4NB	116.2 (17)	C14—C15—C16	121.52 (18)
N5—N4—H4NB	103.2 (17)	N11—C15—C14	118.52 (17)
H4NA—N4—H4NB	106 (2)	C15—C16—C17	118.52 (19)
N5—N4—H4NA	108.2 (16)	C16—C17—C18	124.12 (18)
C2—N5—H5N	117.6 (15)	N12—C17—C18	119.41 (18)
N4—N5—H5N	120.6 (15)	N12—C17—C16	116.47 (19)
H6NA—N6—H6NB	122 (3)	C13—C18—C17	112.38 (17)
C2—N6—H6NA	119 (2)	O16—C18—C17	123.79 (19)
C2—N6—H6NB	119.0 (17)	O16—C18—C13	123.8 (2)
O10—N10—C13	120.59 (16)	C13—C14—H14	120.00
O11—N10—C13	117.81 (17)	C15—C14—H14	120.00
O10—N10—O11	121.59 (18)	C15—C16—H16	121.00
O12—N11—O13	122.5 (2)	C17—C16—H16	121.00
O12—N11—C15	118.43 (19)	N7—C7—C12	118.57 (16)
O13—N11—C15	119.08 (17)	C8—C7—C12	121.73 (18)
O14B—N12—C17	116.3 (10)	N7—C7—C8	119.69 (18)
O15B—N12—C17	113.9 (12)	C7—C8—C9	118.72 (18)
O14A—N12—O15A	115.6 (12)	C8—C9—C10	124.08 (17)
O14B—N12—O15B	117 (2)	N9—C9—C8	116.57 (18)
O14A—N12—C17	118.3 (6)	N9—C9—C10	119.35 (17)
O15A—N12—C17	123.0 (8)	O9A—C10—C11	122.6 (10)
O3—N7—C7	118.38 (18)	C9—C10—C11	112.28 (18)
O4—N7—C7	118.72 (17)	O9B—C10—C9	122.0 (11)
O3—N7—O4	122.90 (19)	O9B—C10—C11	123.0 (7)
O5—N8—C11	117.93 (18)	O9A—C10—C9	123.3 (12)
O6—N8—C11	120.77 (17)	C10—C11—C12	123.67 (19)
O5—N8—O6	121.3 (2)	N8—C11—C12	116.06 (17)
O8—N9—C9	118.53 (18)	N8—C11—C10	120.26 (18)
O7—N9—O8	120.53 (19)	C7—C12—C11	119.44 (17)
O7—N9—C9	120.93 (19)	C9—C8—H8	121.00
S1—C1—N2	122.28 (14)	C7—C8—H8	121.00
S1—C1—N3	119.89 (16)	C7—C12—H12	120.00
N2—C1—N3	117.81 (18)	C11—C12—H12	120.00
S2—C2—N5	123.28 (14)	S4—C5—H5A	109.00
S2—C2—N6	119.36 (16)	S4—C5—H5B	109.00
N5—C2—N6	117.36 (18)	H5A—C5—H5B	109.00
S3—C4A—C4B	91.2 (9)	H5A—C5—H5C	109.00
S3—C4B—C4A	70.1 (9)	S4—C5—H5C	109.00
S3—C3—H3C	109.00	H5B—C5—H5C	109.00
S3—C3—H3A	109.00	S4—C6—H6B	109.00
S3—C3—H3B	109.00	S4—C6—H6C	109.00
H3B—C3—H3C	110.00	S4—C6—H6A	109.00
H3A—C3—H3B	109.00	H6A—C6—H6C	109.00
H3A—C3—H3C	109.00	H6B—C6—H6C	110.00
S3—C4A—H4A1	109.00	H6A—C6—H6B	110.00
			
S2—Zn1—S1—C1	−101.75 (7)	O15A—N12—C17—C18	−1.3 (13)
O1—Zn1—S1—C1	80.25 (8)	O4—N7—C7—C8	−8.4 (3)
N1—Zn1—S1—C1	−14.95 (8)	O3—N7—C7—C8	171.5 (2)
N4—Zn1—S1—C1	−179.13 (8)	O3—N7—C7—C12	−9.7 (3)
S1—Zn1—S2—C2	−94.78 (8)	O4—N7—C7—C12	170.5 (2)
O1—Zn1—S2—C2	83.31 (8)	O6—N8—C11—C10	3.1 (3)
N1—Zn1—S2—C2	−178.29 (8)	O5—N8—C11—C10	−175.7 (2)
N4—Zn1—S2—C2	−15.39 (8)	O5—N8—C11—C12	3.3 (3)
S1—Zn1—O1—S3	41.44 (11)	O6—N8—C11—C12	−177.9 (2)
S2—Zn1—O1—S3	−137.63 (9)	O7—N9—C9—C10	1.3 (3)
N1—Zn1—O1—S3	125.56 (10)	O7—N9—C9—C8	−178.8 (2)
N4—Zn1—O1—S3	−53.34 (11)	O8—N9—C9—C8	1.9 (3)
S1—Zn1—N1—N2	21.71 (13)	O8—N9—C9—C10	−178.1 (2)
S2—Zn1—N1—N2	174.21 (13)	N10—C13—C14—C15	179.42 (18)
O1—Zn1—N1—N2	−86.26 (14)	C14—C13—C18—C17	−2.4 (3)
S1—Zn1—N4—N5	172.48 (13)	N10—C13—C18—O16	−4.8 (3)
S2—Zn1—N4—N5	19.39 (13)	N10—C13—C18—C17	177.75 (18)
O1—Zn1—N4—N5	−78.28 (14)	C18—C13—C14—C15	−0.4 (3)
Zn1—S1—C1—N2	9.21 (17)	C14—C13—C18—O16	175.0 (2)
Zn1—S1—C1—N3	−169.23 (16)	C13—C14—C15—N11	−178.34 (18)
Zn1—S2—C2—N5	13.08 (18)	C13—C14—C15—C16	1.5 (3)
Zn1—S2—C2—N6	−167.45 (16)	N11—C15—C16—C17	−179.62 (19)
C3—S3—O1—Zn1	−89.65 (14)	C14—C15—C16—C17	0.5 (3)
C4A—S3—O1—Zn1	156.5 (2)	C15—C16—C17—N12	176.16 (19)
C4B—S3—O1—Zn1	175.6 (2)	C15—C16—C17—C18	−3.8 (3)
O1—S3—C4A—C4B	88.3 (9)	C16—C17—C18—O16	−172.9 (2)
C3—S3—C4A—C4B	−22.9 (10)	C16—C17—C18—C13	4.6 (3)
O1—S3—C4B—C4A	−96.1 (9)	N12—C17—C18—C13	−175.37 (19)
C3—S3—C4B—C4A	158.3 (9)	N12—C17—C18—O16	7.2 (3)
Zn1—N1—N2—C1	−23.6 (2)	N7—C7—C8—C9	−179.51 (18)
N1—N2—C1—S1	8.4 (3)	C12—C7—C8—C9	1.7 (3)
N1—N2—C1—N3	−173.14 (19)	N7—C7—C12—C11	179.74 (18)
Zn1—N4—N5—C2	−17.8 (2)	C8—C7—C12—C11	−1.4 (3)
N4—N5—C2—S2	1.8 (3)	C7—C8—C9—N9	−179.32 (18)
N4—N5—C2—N6	−177.69 (19)	C7—C8—C9—C10	0.6 (3)
O10—N10—C13—C14	−178.90 (18)	N9—C9—C10—O9A	12.0 (17)
O10—N10—C13—C18	0.9 (3)	N9—C9—C10—C11	177.15 (18)
O11—N10—C13—C14	2.7 (3)	C8—C9—C10—O9A	−168.0 (17)
O11—N10—C13—C18	−177.51 (19)	C8—C9—C10—C11	−2.8 (3)
O12—N11—C15—C14	−11.4 (3)	O9A—C10—C11—N8	−12.7 (18)
O12—N11—C15—C16	168.7 (2)	O9A—C10—C11—C12	168.4 (18)
O13—N11—C15—C14	169.1 (2)	C9—C10—C11—N8	−178.05 (18)
O13—N11—C15—C16	−10.8 (3)	C9—C10—C11—C12	3.0 (3)
O14A—N12—C17—C16	19.5 (17)	N8—C11—C12—C7	179.91 (18)
O14A—N12—C17—C18	−160.6 (17)	C10—C11—C12—C7	−1.1 (3)
O15A—N12—C17—C16	178.7 (12)		

### Hydrogen-bond geometry (Å, °)

**Table:**

*D*—H···*A*	*D*—H	H···*A*	*D*···*A*	*D*—H···*A*
N1—H1NA···O1W^i^	0.87 (2)	2.20 (2)	2.974 (3)	148 (2)
N2—HN2···O15A^i^	0.80 (2)	2.41 (3)	3.01 (2)	133 (2)
N2—HN2···O16^i^	0.80 (2)	1.96 (2)	2.693 (2)	152 (2)
N3—H3NA···O10^i^	0.87 (3)	2.44 (3)	3.166 (2)	141 (2)
N3—H3NA···O16^i^	0.87 (3)	2.03 (3)	2.790 (3)	146 (3)
N3—H3NB···O5^ii^	0.77 (2)	2.24 (2)	3.004 (3)	173 (2)
N5—H5N···O7^iii^	0.84 (2)	2.37 (2)	3.007 (3)	133 (2)
N5—H5N···O9A^iii^	0.84 (2)	1.94 (3)	2.698 (17)	150 (2)
N4—H4NA···O2^iii^	0.88 (2)	2.20 (2)	2.933 (3)	141 (2)
N4—H4NB···S1^iv^	0.84 (3)	2.63 (3)	3.457 (2)	170 (2)
N6—H6NA···O6^iii^	0.82 (3)	2.41 (3)	3.081 (3)	140 (2)
N6—H6NA···O9A^iii^	0.82 (3)	2.05 (5)	2.76 (3)	145 (3)
N6—H6NB···O11^v^	0.82 (3)	2.22 (3)	3.034 (3)	172 (3)
O1W—H1WA···O8	0.819 (19)	2.27 (2)	3.059 (3)	163 (3)
O1W—H1WB···O2	0.80 (2)	2.02 (2)	2.806 (3)	171 (3)
C6—H6B···O2^iii^	0.96	2.52	3.347 (3)	145
C8—H8···O4^vi^	0.93	2.47	3.390 (3)	168
C12—H12···O5	0.93	2.31	2.636 (3)	100
C14—H14···O11	0.93	2.30	2.631 (3)	101
C4A—H4A2···O1W	0.96	2.48	3.430 (7)	170

Symmetry codes: (i) *x*, *y*−1, *z*; (ii) −*x*, −*y*+1, −*z*; (iii) −*x*, −*y*+1, −*z*+1; (iv) −*x*, −*y*, −*z*+1; (v) *x*, *y*−1, *z*+1; (vi) −*x*+1, −*y*+1, −*z*.
